# A Fluorescent and Magnetic Hybrid Tracer for Improved Sentinel Lymphadenectomy in Prostate Cancer Patients

**DOI:** 10.3390/biomedicines11102779

**Published:** 2023-10-13

**Authors:** Svenja Engels, Bianca Michalik, Lena Dirks, Matthias N. van Oosterom, Friedhelm Wawroschek, Alexander Winter

**Affiliations:** 1 University Hospital for Urology, Klinikum Oldenburg, Department of Human Medicine, School of Medicine and Health Sciences, Carl von Ossietzky University Oldenburg, 26129 Oldenburg, Germany; engels.svenja@klinikum-oldenburg.de (S.E.); michalik.bianca@klinikum-oldenburg.de (B.M.); lena.dirks@uni-oldenburg.de (L.D.); wawroschek.friedhelm@klinikum-oldenburg.de (F.W.); 2 Interventional Molecular Imaging Laboratory, Department of Radiology, Leiden University Medical Center, 2300 RC Leiden, The Netherlands; m.n.van_oosterom@lumc.nl

**Keywords:** hybrid, fluorescence, magnetic tracer, sentinel lymph node, prostate cancer

## Abstract

In prostate cancer, sentinel lymph node dissection (sLND) offers a personalized procedure with staging ability which is at least equivalent to extended LND while inducing lower morbidity. A bimodal fluorescent–radioactive approach was introduced to improve sentinel LN (SLN) detection. We present the first in-human case series on exploring the use of a fluorescent–magnetic hybrid tracer in a radiation-free sLND procedure. Superparamagnetic iron oxide nanoparticles and indocyanine green were administered simultaneously in five prostate cancer patients scheduled for extended LND, sLND and radical prostatectomy. In situ and ex vivo fluorescence and magnetic signals were documented for each LN sample detected via a laparoscopic fluorescence imaging and magnetometer system. Fluorescence and magnetic activity could be detected in all patients. Overall, 19 lymph node spots could be detected in situ, 14 of which were fluorescently active and 18 of which were magnetically active. In two patients, no fluorescent LNs could be detected in situ. The separation of the LN samples resulted in a total number of 30 SLNs resected. Ex vivo measurements confirmed fluorescence in all but two magnetically active SLNs. One LN detected in situ with both modalities was subsequently shown to contain a metastasis. This study provides the first promising results of a bimodal, radiation-free sLND, combining the advantages of both the magnetic and fluorescence approaches.

## 1. Introduction

In prostate cancer, in addition to the relevance of the prostate-specific antigen (PSA) value for diagnosis [1], the lymph node (LN) status is a relevant prognostic factor for the oncological outcome, as well as for planning adjuvant therapy [2]. LN metastasis occurs in 3–80% of prostate cancer patients undergoing radical prostatectomy [3]. Despite the rise of new imaging techniques, such as prostate-specific membrane antigen (PSMA) PET/CT [4,5], extended lymph node dissection (eLND) remains the gold standard for LN staging in clinically localized prostate cancer [6]. However, while the invasiveness and the risk of associated complications and/or morbidity increase with the number of LNs removed [2,7], the prevalence of LN involvement directly correlates with the number of dissected LNs and/or the extent of LND [8,9]. Furthermore, there are indications that the dissection of LN metastases as part of pelvic LND (PLND) has therapeutic benefit, especially for patients with minimal lymph node invasion (LNI) [10,11,12]. In this context, sentinel lymph node (SLN) dissection (sLND) was established in prostate cancer to reduce the number of dissected LNs without a loss of sensitivity as it enables the surgeon to selectively remove the LNs which drain directly from the prostate and thus bear the greatest risk of harboring metastasis [13,14,15].

There is accumulating evidence that a fluorescent–radioactive hybrid tracer outperforms other conventional tracers in its nodal staging ability [16,17]. Furthermore, using a fluorescent–radioactive hybrid tracer during sLND to label SLNs may be associated with lower rates of biochemical recurrence than eLND and with low rates of complications as well [18,19]. This bimodal approach for the detection of SLNs seems especially advantageous for minimally invasive and robot-assisted surgery [20], but it has the disadvantages of depending on the availability of nuclear medicine and exposing patients and surgical staff to radiation. Magnetic tracers consisting of superparamagnetic iron oxide nanoparticles (SPIONs) were introduced as radiation-free alternatives to conventional radiotracers in various tumor entities including breast cancer and prostate cancer [21,22]. In a recent analysis of data from 3000 patients, our group showed equally high levels of reliability of both the radiotracer and the magnetic tracer in detecting LN metastasis [23]. Unfortunately, laparoscopic magnetometer probes for the SPION-labelled detection of SLNs are currently being developed but are not yet approved for routine clinical use [24,25,26]. Analogous to the fluorescent–radioactive hybrid tracer, additional fluorescence labeling of the SPION tracer could provide a further benefit for SLN detection, especially in minimally invasive robotic surgery. To our knowledge, there are only a few pre-clinical studies with fluorescent–magnetic hybrid tracers which show the general feasibility of fluorescent–magnetic SLN detection in animal models and human skin tissue explants [25,27,28,29,30]. In this small case series (according to IDEAL Stage 2a [31]), we therefore introduce the first human in situ implementation of a fluorescent–magnetic hybrid tracer for radiation-free bimodal sLND during radical prostatectomy.

## 2. Materials and Methods

### 2.1. Patients

This case series included five prostate cancer patients who were scheduled for a radical prostatectomy in combination with both extended pelvic LND and sLND at the University Hospital for Urology Oldenburg, Germany, in 07/2022. The patients did not undergo any pre-treatment that might have altered the lymphatic drainage of the prostate, such as anti-hormonal therapy or transurethral prostate surgery. All patients were informed verbally and in writing about radical prostatectomy, magnetometer-guided sLND and the use of indocyanine green (ICG) for LN visualization. All patients signed a consent form regarding the use of their clinical data in a scientific publication.

### 2.2. Tracer

We used a commercially available magnetic tracer (Magtrace, Endomagnetics Ltd., Cambridge, UK) consisting of superparamagnetic iron oxide nanoparticles (SPIONs) coated with carbodextran and dissolved in saline. To synthesize the fluorescent–magnetic hybrid tracer, either 50 µL or 100 µL of 5 mg/mL ICG (Verdye, Diagnostic Green GmbH, Aschheim-Dornach, Germany; dissolved in water for injection) was added to 2 mL of the SPION tracer, similar to the procedure described before [27]. To test for the effects of concentration on fluorescence detection in situ, we applied 50 µL of the fluorescent tracer (i.e., a total amount of 0.25 mg of ICG) in the first three patients and raised this to 100 µL (i.e., a total amount of 0.5 mg of ICG) in the other two patients.

### 2.3. Surgery

The day before surgery (median 19.3 h, IQR 18.6–21.6 h), a urologist transrectally injected the fluorescent–magnetic hybrid tracer under ultrasound guidance into each lobe of the prostate in three deposits, respectively [22]. PLND was performed during open retropubic surgery. The lymphatic drainage area of the prostate was searched for fluorescent LNs via a near-infrared camera (IMAGE1 S™ Rubina^®^, Karl Storz SE & Co. KG, Tuttlingen, Germany) and for magnetic LNs via a handheld magnetometer probe (Sentimag^®^, Endomagnetics Ltd., Cambridge, UK). All LN spots identified in situ via either mode were selectively surgically removed. As recommended by the European guidelines on prostate cancer [6], PLND was completed by bilateral eLND according to the standard anatomical template. The lymphatic tissue samples from the LN spots detected in situ LN spots could frequently be separated into several single LNs ex vivo, resulting in a higher number of SLNs measured ex vivo. All LNs as well as the radical prostatectomy specimens were checked for ex vivo fluorescence and magnetic activity, respectively. After surgery, all tissue samples were fixed in formalin for approximately 24 h and were then routinely processed. For each tissue sample, hematoxylin–eosin stains were microscopically analyzed for tumor infiltration by a pathologist experienced in uropathology.

## 3. Results

The clinical and histopathological characteristics of the five patients and the details of the tracer injection and in situ detection data are summarized in Table 1.

**Figure 1:**
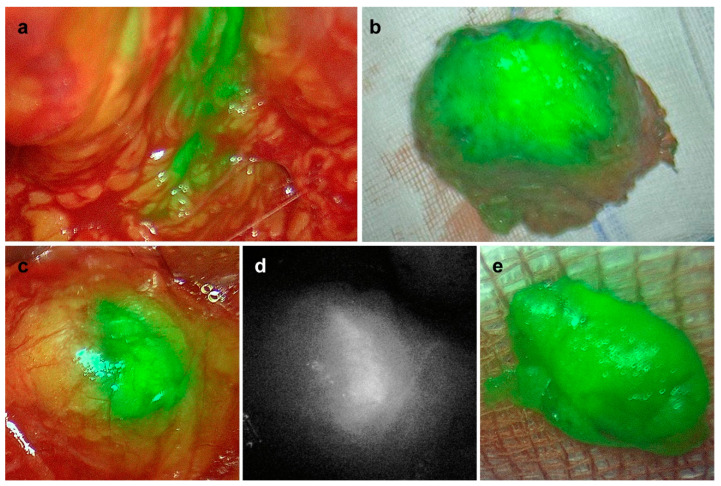
In vivo (**a**,**c**,**d**) and ex vivo (**b**,**e**) detection of fluorescence in the prostate (**a**,**b**) and in one lymph node (**c**–**e**) of patient #4. (**a**–**c**,**e**) Overlay; (**d**) monochromatic.

In all five patients, we could detect magnetic activity and fluorescence. In the prostates of four of the five patients, we could detect magnetic activity as well as fluorescence (Table 1). In patient #2, we were not able to measure the fluorescence of the prostate in situ as the fluorescence camera was not ready to use at this time during the surgery. LN samples could be located in all patients via magnetic activity and/or fluorescence (Table 1). Figure 1 shows the in situ and ex vivo fluorescence camera images of the prostate, as well as of one LN of patient #4.

All histopathologically confirmed LNs detected via fluorescence were also magnetically active. One fluorescent-only LN sample turned out to be lymphatic fatty tissue upon histopathological examination (indicated by ** in Table 1). In two of the three patients with the lower concentrations of ICG, we could only detect magnetically labeled but non-fluorescent LNs in situ (patients #2 and #3, Table 1). In one of these two patients, ex vivo measurements showed fluorescence in all magnetically active LNs as well (patient #3, Table 1). In the other patients, two of the SLNs were magnetically active only (patient #2, Table 1). It should be noted that the number of detected SLN samples in situ (*n* = 19) is not equivalent to the total number of SLNs identified via histopathology (*n* = 30) (Table 1), as a detailed ex vivo investigation may reveal several single LNs in one dissected LN tissue sample. Overall, there was a high level of accordance between the two modalities, with 28 of 30 SLNs being both magnetically active and fluorescent and the remaining two found to be only magnetically active. A histopathological examination revealed the metastasis of one LN in an SLN of one patient (patient #1, Table 1). The metastatic SLN was detected in situ via both detection modalities. An examination of the 20 additional LNs removed during ePLND revealed no further metastases.

## 4. Discussion

This study represents the first in-human description of the in situ administration of an ICG-SPION hybrid tracer for LN visualization in prostate cancer patients. Our data show the principal applicability of this hybrid tracer that allows for the magnetic and fluorescent in situ identification of SLNs draining directly from the prostate.

Applying the ICG-SPION hybrid tracer in prostate cancer patients, we observed magnetic activity in SLNs comparable to use of the traditional SPION tracer only. Despite the very small sample size in our study, the number of SLNs, the overall number of LNs removed and the number of LN metastases are comparable to prior studies and data collected in our center using the magnetic tracer alone [23]. Therefore, the addition of ICG does not seem to impair the magnetic tracer or influence the lymphatic drainage or accumulation of the magnetic tracer within the LNs. This is also in line with the results of animal experiments using different SPION-ICG applications [25,27,28,29,30].

ICG alone has been used as fluorescent lymphangiographic agent, labeling LNs in various tumor applications such as prostate cancer [32], breast cancer [33], uterine cancer [34], cervical cancer [35] and gastric cancer [36]. Since it is non-toxic at doses below 2 mg/kg body weight, has a low rate of adverse reactions [37] and is highly reliable, ICG has been recommended for SLN visualization, e.g., in endometrial cancer, by several international guidelines [38]. While the intraoperative use of ICG provides the advantage of tracking lymphatic pathways in real time, it is not specific to SLNs [39], and its limited penetration depth (<1.5 cm [40]) makes it a superficial technique, lacking an option for preoperative imaging. As such, relying on fluorescence imaging only might complicate the identification of SLNs depending on their anatomical location [41].

In prostate cancer, a fluorescent–radioactive hybrid tracer [16] therefore seems to be a suitable alternative for SLN tracking [17]. The larger molecular size of the tracer complex compared to “free” ICG slows down the drain and leads to greater accumulation within the SLNs (i.e., increased SLN specificity), allowing for tracer injection to be carried out one day preoperatively and the preoperative localization of the SLNs via imaging, which enhances intraoperative detection. This bimodal approach combines the advantages of the approved radio-guided SLN procedure, including preoperative lymphatic mapping with an intraoperative radioactive and/or fluorescence confirmation of the localization of the SLNs [20]. In our center, we use a magnetic SPION tracer as a reliable and radiation-free alternative to radioisotope SLN labeling. It was first introduced in breast cancer [21,42,43] and later on in several other tumor entities, including prostate and penile cancer [22,23,44]. This technique also allows for the pre-operative mapping of magnetically labelled SLNs through magnetic resonance imaging comparable to scintigraphy in radioactive marking [45]. As an additional advantage, the fluorescent signal also allows for the visual identification of SLNs, e.g., close to the prostate, where it is not possible to differentiate between the magnetic signals of the prostate and the SLNs. While the use of pure ICG for LND might cause the problem of leakage into the surgical field [46], this seems not to be the case when using hybrid tracers like our fluorescent–magnetic tracer or an ICG-99mTc-nanocolloid [17] for LNDs, probably because of the larger molecular size.

The development of a fluorescent–magnetic hybrid tracer is therefore the next logical step toward optimizing the detection of SLNs, especially for minimally invasive and robotic PLND during radical prostatectomy. After our own successful animal studies in which we combined the magnetic tracer that we normally use in our clinical routine with ICG, its use in this small case series was then required to achieve this purpose.

## 5. Conclusions

Our study presents the first promising results of radiation-free bimodal SLN visualization in prostate cancer patients. Future studies need to determine the optimal concentration of ICG within the SPION tracer and test the reliability of the fluorescent–magnetic hybrid tracer with larger numbers of patients, including preoperative visualization via MRI.

## Figures and Tables

**Table 1:** Clinical and histopathological patient characteristics and in situ detection data.

Patient	#1	#2	#3	#4	#5	Overall
Clinical information						
Age [years]	62	70	69	67	70	
BMI	24	24	22	27	23	
Total PSA [µg/L] ^†^	4.4	2.8	19.1	2.3	5.9	
Clinical tumor category	2	2	3	1c	2	
Biopsy ISUP grade	4	4	3	1	2	
Positive biopsy cores [%]	7	58	29	17	21	
Risk of LNI ^††^ [%]	34	63	64	3	12	
Tracer injection						
Total amount of ICG [mg]	0.25	0.25	0.25	0.5	0.5	
Time injection to surgery [h]	18.6	22.4	19.3	16.8	21.6	
In situ detection						
Prostate magnetic activity	yes	yes	yes	yes	yes	
Prostate fluorescence	yes	n.a. *	yes	yes	yes	
No. of lymph node spots	3	2	2	7	5	19 **
No. of magnetically active spots	3	2	2	7	4	18
No. of fluorescent spots	2	0	0	7	5 **	14
Ex vivo detection						
Prostate magnetic activity	yes	yes	yes	yes	yes	
Prostate fluorescence	yes	yes	yes	yes	yes	
No. of LNs resected	13	8	12	8	9	50
No. of SLNs resected	5	6	5	7	7	30
No. of magnetically active SLNs	5	6	5	7	7	30
No. of fluorescent SLNs	5	4	5	7	7 **	28
Post-operative information						
Pathological tumor category	2c	3b	2c	2c	3a	
Post-operative ISUP grade	5	5	3	2	2	
pN	1	0	0	0	0	

^†^ Total PSA values were determined in our laboratory using electrochemiluminescence immunoassay (Elecsys^®^ total PSA, Roche Diagnostics GmbH, Mannheim, Germany) using a cobas^®^ e801 analytical unit (Roche Diagnostics GmbH, Mannheim, Germany); ^††^ as calculated from our nomogram [3]; * no in situ measurement as the fluorescence camera was not yet ready to use; ** one fluorescent lymph node sample turned out to be lymphatic fatty tissue upon histopathological examination. BMI: body mass index; ICG: indocyanine green; ISUP: International Society of Urological Pathology; LNI: lymph node involvement; *n*: number; pN: pathological nodal status; PSA: prostate-specific antigen; SLNs: sentinel lymph nodes.

## Data Availability

The data presented in this study are available upon request from the corresponding author.

## References

[1] Ferraro S., Biganzoli D., Rossi R.S., Palmisano F., Bussetti M., Verzotti E., Gregori A., Bianchi F., Maggioni M., Ceriotti F. (2023). Individual risk prediction of high grade prostate cancer based on the combination between total prostate-specific antigen (PSA) and free to total PSA ratio. Clin. Chem. Lab. Med..

[2] Fossati N., Willemse P.-P.M., Van den Broeck T., van den Bergh R.C.N., Yuan C.Y., Briers E., Bellmunt J., Bolla M., Cornford P., De Santis M. (2017). The benefits and harms of different extents of lymph node dissection during radical prostatectomy for prostate cancer: A systematic review. Eur. Urol..

[3] Winter A., Kneib T., Wasylow C., Reinhardt L., Henke R.-P., Engels S., Gerullis H., Wawroschek F. (2017). Updated nomogram incorporating percentage of positive cores to predict probability of lymph node invasion in prostate cancer patients undergoing sentinel lymph node dissection. J. Cancer.

[4] Hope T.A., Eiber M., Armstrong W.R., Juarez R., Murthy V., Lawhn-Heath C., Behr S.C., Zhang L., Barbato F., Ceci F. (2021). Diagnostic accuracy of 68Ga-PSMA-11 PET for pelvic nodal metastasis detection prior to radical prostatectomy and pelvic lymph node dissection: A multicenter prospective phase 3 imaging trial. JAMA Oncol..

[5] Klingenberg S., Jochumsen M.R., Ulhøi B.P., Fredsøe J., Sørensen K.D., Borre M., Bouchelouche K. (2021). 68Ga-PSMA PET/CT for primary lymph node and distant metastasis NM staging of high-risk prostate cancer. J. Nucl. Med..

[6] Mottet N., van den Bergh R.C.N., Briers E., Van den Broeck T., Cumberbatch M.G., De Santis M., Fanti S., Fossati N., Gandaglia G., Gillessen S. (2021). EAU-EANM-ESTRO-ESUR-SIOG Guidelines on Prostate Cancer—2020 Update. Part 1: Screening, Diagnosis, and Local Treatment with Curative Intent. Eur. Urol..

[7] Briganti A., Chun F.K.H., Salonia A., Suardi N., Gallina A., Da Pozzo L.F., Roscigno M., Zanni G., Valiquette L., Rigatti P. (2006). Complications and other surgical outcomes associated with extended pelvic lymphadenectomy in men with localized prostate cancer. Eur. Urol..

[8] Heidenreich A., Ohlmann C.H., Polyakov S. (2007). Anatomical extent of pelvic lymphadenectomy in patients undergoing radical prostatectomy. Eur. Urol..

[9] Briganti A., Blute M.L., Eastham J.H., Graefen M., Heidenreich A., Karnes J.R., Montorsi F., Studer U.E. (2009). Pelvic lymph node dissection in prostate cancer. Eur. Urol..

[10] Choo M.S., Kim M., Ku J.H., Kwak C., Kim H.H., Jeong C.W. (2017). Extended versus standard pelvic lymph node dissection in radical prostatectomy on oncological and functional outcomes: A systematic review and meta-analysis. Ann. Surg. Oncol..

[11] Seiler R., Studer U.E., Tschan K., Bader P., Burkhard F.C. (2014). Removal of limited nodal disease in patients undergoing radical prostatectomy: Long-term results confirm a chance for cure. J. Urol..

[12] Winter A., Henke R.-P., Wawroschek F. (2015). Targeted salvage lymphadenectomy in patients treated with radical prostatectomy with biochemical recurrence: Complete biochemical response without adjuvant therapy in patients with low volume lymph node recurrence over a long-term follow-up. BMC Urol..

[13] Wawroschek F., Vogt H., Wengenmair H., Weckermann D., Hamm M., Keil M., Graf G., Heidenreich P., Harzmann R. (2003). Prostate lymphoscintigraphy and radio-guided surgery for sentinel lymph node identification in prostate cancer. Urol. Int..

[14] Wit E.M.K., Acar C., Grivas N., Yuan C., Horenblas S., Liedberg F., Valdes Olmos R.A., van Leeuwen F.W.B., van den Berg N.S., Winter A. (2017). Sentinel node procedure in prostate cancer: A systematic review to assess diagnostic accuracy. Eur. Urol..

[15] de Korne C.M., Wit E.M., de Jong J., Valdés Olmos R.A., Buckle T., van Leeuwen F.W.B., van der Poel H.G. (2019). Anatomical localization of radiocolloid tracer deposition affects outcome of sentinel node procedures in prostate cancer. Eur. J. Nucl. Med. Mol. Imaging.

[16] van der Poel H.G., Buckle T., Brouwer O.R., Valdés Olmos R.A., van Leeuwen F.W. (2011). Intraoperative laparoscopic fluorescence guidance to the sentinel lymph node in prostate cancer patients: Clinical proof of concept of an integrated functional imaging approach using a multimodal tracer. Eur. Urol..

[17] KleinJan G.H., van Werkhoven E., van den Berg N.S., Karakullukcu M.B., Zijlmans H., van der Hage J.A., van de Wiel B.A., Buckle T., Klop W.M.C., Horenblas S. (2018). The best of both worlds: A hybrid approach for optimal pre- and intraoperative identification of sentinel lymph nodes. Eur. J. Nucl. Med. Mol. Imaging.

[18] Grivas N., Wit E.M.K., Kuusk T., KleinJan G.H., Donswijk M.L., van Leeuwen F.W.B., van der Poel H.G. (2018). The impact of adding sentinel node biopsy to extended pelvic lymph node dissection on biochemical recurrence in prostate cancer patients treated with robot-assisted radical prostatectomy. J. Nucl. Med..

[19] Mazzone E., Dell’Oglio P., Grivas N., Wit E., Donswijk M., Briganti A., Leeuwen F.V., van der Poel H. (2021). Diagnostic value, oncologic outcomes, and safety profile of image-guided surgery technologies during robot-assisted lymph node dissection with sentinel node biopsy for prostate cancer. J. Nucl. Med..

[20] van Leeuwen F.W.B., Winter A., van Der Poel H.G., Eiber M., Suardi N., Graefen M., Wawroschek F., Maurer T. (2019). Technologies for image-guided surgery for managing lymphatic metastases in prostate cancer. Nat. Rev. Urol..

[21] Douek M., Klaase J., Monypenny I., Kothari A., Zechmeister K., Brown D., Wyld L., Drew P., Garmo H., Agbaje O. (2014). Sentinel node biopsy using a magnetic tracer versus standard technique: The SentiMAG multicentre trial. Ann. Surg. Oncol..

[22] Winter A., Woenckhaus J., Wawroschek F. (2014). A novel method for intraoperative sentinel lymph node detection in prostate cancer patients using superparamagnetic iron oxide nanoparticles and a handheld magnetometer: The initial clinical experience. Ann. Surg. Oncol..

[23] Engels S., Michalik B., Meyer L.-M., Nemitz L., Wawroschek F., Winter A. (2021). Magnetometer-guided sentinel lymph node dissection in prostate cancer: Rate of lymph node involvement compared with radioisotope marking. Cancers.

[24] Kuwahata A., Tanaka R., Matsuda S., Amada E., Irino T., Mayanagi S., Chikaki S., Saito I., Tanabe N., Kawakubo H. (2020). Development of magnetic probe for sentinel lymph node detection in laparoscopic navigation for gastric cancer patients. Sci. Rep..

[25] Mihara K., Matsuda S., Nakamura Y., Aiura K., Kuwahata A., Chikaki S., Sekino M., Kusakabe M., Suzuki S., Fuchimoto D. (2021). Intraoperative laparoscopic detection of sentinel lymph nodes with indocyanine green and superparamagnetic iron oxide in a swine gallbladder cancer model. PLoS ONE.

[26] van de Loosdrecht M.M., Molenaar L., Krooshoop E.J.G., ten Haken B., Meijerink W.J.H.J., Alic L., Broeders I.A.M.J. (2021). Laparoscopic Probe for Sentinel Lymph Node Harvesting Using Magnetic Nanoparticles. IEEE Trans. Biomed. Eng..

[27] Azargoshasb S., Molenaar L., Rosiello G., Buckle T., van Willigen D.M., van de Loosdrecht M.M., Welling M.M., Alic L., van Leeuwen F.W.B., Winter A. (2022). Advancing intraoperative magnetic tracing using 3D freehand magnetic particle imaging. Int. J. Comput. Assist. Radiol. Surg..

[28] Cousins A., Krishnan S., Krishnan G., Pham N., Milanova V., Nelson M., Shetty A., Ikoma N., Thierry B. (2023). Preclinical evaluation of sentinel node localization in the stomach via mannose-labelled magnetic nanoparticles and indocyanine green. Surg. Endosc..

[29] Krishnan G., Cousins A., Pham N., Milanova V., Nelson M., Krishnan S., van den Berg N.S., Shetty A., Rosenthal E.L., Wormald P.J. (2022). Preclinical feasibility of robot-assisted sentinel lymph node biopsy using multi-modality magnetic and fluorescence guidance in the head and neck. Head Neck.

[30] Kuwahata A., Ahmed M., Saeki K., Chikaki S., Kaneko M., Qiu W., Xin Z., Yamaguchi S., Kaneko A., Douek M. (2018). Combined use of fluorescence with a magnetic tracer and dilution effect upon sentinel node localization in a murine model. Int. J. Nanomed..

[31] Marcus H.J., Bennett A., Chari A., Day T., Hirst A., Hughes-Hallett A., Kolias A., Kwasnicki R.M., Martin J., Rovers M. (2022). IDEAL-D Framework for Device Innovation: A Consensus Statement on the Preclinical Stage. Ann. Surg..

[32] Yuen K., Miura T., Sakai I., Kiyosue A., Yamashita M. (2015). Intraoperative Fluorescence Imaging for Detection of Sentinel Lymph Nodes and Lymphatic Vessels during Open Prostatectomy using Indocyanine Green. J. Urol..

[33] Goonawardena J., Yong C., Law M. (2020). Use of indocyanine green fluorescence compared to radioisotope for sentinel lymph node biopsy in early-stage breast cancer: Systematic review and meta-analysis. Am. J. Surg..

[34] Eriksson A.G., Beavis A., Soslow R.A., Zhou Q., Abu-Rustum N.R., Gardner G.J., Zivanovic O., Long Roche K., Sonoda Y., Leitao M.M. (2017). A Comparison of the Detection of Sentinel Lymph Nodes Using Indocyanine Green and Near-Infrared Fluorescence Imaging Versus Blue Dye During Robotic Surgery in Uterine Cancer. Int. J. Gynecol. Cancer.

[35] Ruscito I., Gasparri M.L., Braicu E.I., Bellati F., Raio L., Sehouli J., Mueller M.D., Panici P.B., Papadia A. (2016). Sentinel Node Mapping in Cervical and Endometrial Cancer: Indocyanine Green Versus Other Conventional Dyes—A Meta-Analysis. Ann. Surg. Oncol..

[36] Belia F., Biondi A., Agnes A., Santocchi P., Laurino A., Lorenzon L., Pezzuto R., Tirelli F., Ferri L., D’Ugo D. (2022). The Use of Indocyanine Green (ICG) and Near-Infrared (NIR) Fluorescence-Guided Imaging in Gastric Cancer Surgery: A Narrative Review. Front. Surg..

[37] Hope-Ross M., Yannuzzi L.A., Gragoudas E.S., Guyer D.R., Slakter J.S., Sorenson J.A., Krupsky S., Orlock D.A., Puliafito C.A. (1994). Adverse reactions due to indocyanine green. Ophthalmology.

[38] Dick A., Perri T., Kogan L., Brandt B., Meyer R., Levin G. (2023). Sentinel lymph node mapping in endometrial cancer: A comparison of main national and international guidelines. Int. J. Gynaecol. Obstet..

[39] Wit E.M.K., KleinJan G.H., Berrens A.-C., van Vliet R., van Leeuwen P.J., Buckle T., Donswijk M.L., Bekers E.M., van Leeuwen F.W.B., van der Poel H.G. (2023). A hybrid radioactive and fluorescence approach is more than the sum of its parts; outcome of a phase II randomized sentinel node trial in prostate cancer patients. Eur. J. Nucl. Med. Mol. Imaging.

[40] Stoffels I., Dissemond J., Pöppel T., Schadendorf D., Klode J. (2015). Intraoperative Fluorescence Imaging for Sentinel Lymph Node Detection: Prospective Clinical Trial to Compare the Usefulness of Indocyanine Green vs Technetium Tc 99m for Identification of Sentinel Lymph Nodes. JAMA Surg..

[41] Meershoek P., Buckle T., van Oosterom M.N., KleinJan G.H., van der Poel H.G., van Leeuwen F.W.B. (2020). Can Intraoperative Fluorescence Imaging Identify All Lesions While the Road Map Created by Preoperative Nuclear Imaging Is Masked?. J. Nucl. Med..

[42] Ahmed M., de Rosales R.T.M., Douek M. (2013). Preclinical studies of the role of iron oxide magnetic nanoparticles for nonpalpable lesion localization in breast cancer. J. Surg. Res..

[43] Thill M., Kurylcio A., Welter R., van Haasteren V., Grosse B., Berclaz G., Polkowski W., Hauser N. (2014). The Central-European SentiMag study: Sentinel lymph node biopsy with superparamagnetic iron oxide (SPIO) vs. radioisotope. Breast.

[44] Winter A., Kowald T., Engels S., Wawroschek F. (2020). Magnetic resonance sentinel lymph node imaging and magnetometer-guided intraoperative detection in penile cancer, using superparamagnetic iron oxide nanoparticles: First results. Urol. Int..

[45] Winter A., Kowald T., Paulo T.S., Goos P., Engels S., Gerullis H., Schiffmann J., Chavan A., Wawroschek F. (2018). Magnetic resonance sentinel lymph node imaging and magnetometer-guided intraoperative detection in prostate cancer using superparamagnetic iron oxide nanoparticles. Int. J. Nanomed..

[46] Manny T.B., Patel M., Hemal A.K. (2014). Fluorescence-enhanced robotic radical prostatectomy using real-time lymphangiography and tissue marking with percutaneous injection of unconjugated indocyanine green: The initial clinical experience in 50 patients. Eur. Urol..

